# Preterm infants with positive conjunctival swab culture: risk factors and association with late-onset sepsis–a retrospective cohort study

**DOI:** 10.3389/fped.2023.1259558

**Published:** 2023-11-15

**Authors:** Ashraf Gad, Amr Khalil, Muhammed Halil, Prem Chandra, Aly Soliman, Abdel Rahman E’mar, Marwa Ibrahim, Fadi Al Khzzam, Talal AlHendawi, Manal Hamed, Mohammad A. A. Bayoumi, Hawabibee Petkar

**Affiliations:** ^1^ Neonatal Intensive Care Unit, Women’s Wellness and Research Center, Hamad Medical Corporation, Doha, Qatar; ^2^ Pediatric Department, Weill Cornell Medicine, Doha, Qatar; ^3^ Division of Neonatology, London Health Science, Western University, London, ON, Canada; ^4^ Medical Research Center, Hamad Medical Corporation, Doha, Qatar; ^5^ Deparment of Pediatrics, SUNY Upstate Medical University, Syracuse, NY, United States; ^6^ Department of Pediatrics, Cleveland Clinic Children’s, Cleveland, OH, United States; ^7^ Department of Pediatrics, Hamad Medical Corporation, Doha, Qatar; ^8^ Microbiology Division, Department of Laboratory Medicine and Pathology, Hamad Medical Corporation, Doha, Qatar

**Keywords:** neonatal sepsis, preterm, neonatal bacteremia, conjunctivitis, conjunctival swab, conjunctival culture, eye discharge

## Abstract

**Introduction:**

Purulent conjunctival discharge in hospitalized preterm infants may indicate conjunctivitis and warrant treatment. The purpose of this study was to examine the relationship between positive conjunctival swab (CS) culture and late-onset sepsis (LOS) in preterm infants.

**Methods:**

A retrospective cohort study was conducted to determine the relationship between positive CS culture growth results (CSP) obtained in preterm infants ≤34 weeks' gestation and the development of LOS within 120 h of obtaining CS compared with those who had negative CS culture results (CSN). Electronic medical records were reviewed from January 2015 until December 2019 for preterm infants presenting with purulent conjunctival discharge and underwent CS culture testing due to suspected conjunctivitis.

**Results:**

Of the 234 CS cultures obtained during the study period, 145 (61.9%) were CSP compared to 89 (38.1%) CSN cultures. Gram-negative organisms accounted for 70% of all CSP cultures, with the remaining 30% being Gram-positive. Patients with CSP were smaller, younger, had lower 1-minute APGAR scores, and required respiratory support more frequently than those with CSN. Infants with CSP received antibiotics for longer periods, both topically and systemically. Infants who developed LOS were more likely to require invasive ventilation (adjusted odds ratio, 33.5; 95% CI, 2.52–446.5, *p *= 0.008). The incidence of LOS between the two groups was similar, with 6.2% observed in the CSP group compared to 3.4% in the CSN group (*p *= 0.543). Similarly, the rates of bacteremia were similar in both groups. Of the CSP patients who were presented with bacteremia, four out of seven (57%) exhibited bacteremia caused by the same organism found in their CS cultures. Similarly, within the entire cohort, respiratory cultures were performed on nine intubated patients within two weeks of obtaining CS cultures. Of these, in the CSP group, five out of six (83%) showed an organism identical to that found in the CS cultures.

**Conclusion:**

The study found a significant proportion of positive CS cultures in preterm infants, with distinct patient characteristics and treatment compared to negative cultures. While the incidence of LOS was not significantly different between the two groups, some CSP patients demonstrated bacteremia with the same CS organism, suggesting a possible connection between conjunctival or respiratory colonization and bacteremia.

## Introduction

Conjunctivitis is a serious infection affecting up to 5% of neonates in the neonatal intensive care unit (NICU) (1), with the incidence of pathogenic bacteria in the conjunctivae increasing to 14.5% in surveillance studies (2). Hospitalized preterm infants are at a particularly high risk due to their naive immune system, immature lacrimal duct systems, prolonged hospitalization, respiratory support, invasive procedures, and colonization of conjunctivae by respiratory organisms during neonatal care (1). Conjunctival discharge is a common finding in preterm infants, particularly in the NICU. Although it can be caused by non-infectious factors like irritation or lacrimal duct blockage, it often indicates conjunctivitis when accompanied by erythema or eye edema (3). However, physical findings may not always be present in preterm infants, potentially leading to under-diagnosis of clinically significant conjunctivitis in this vulnerable population (2). Furthermore, the National Nosocomial Infection Surveillance (NNIS) definition of conjunctivitis may miss up to 38% of clinical hospital-acquired conjunctivitis in neonates (1). Treatment often involves conservative or empirical use of topical antimicrobial agents, especially if other conjunctivitis signs are absent or culture grows polymorphic or skin flora such as coagulase-negative staphylococci (CoNS). Some neonatologists may also administer topical and, in some instances, systemic antimicrobials regardless of systemic infection signs.

Whether considered colonization or active infection, the pathogens isolated could serve as a potential source of sepsis or bacteremia in vulnerable preterm and low birth weight infants, particularly those on non-invasive ventilation, and may also result in life-threatening systemic infections. Many of these hospital-acquired bacteria, such as *Escherichia coli* (*E. coli*), are invasive pathogens resistant to antimicrobials (1, 2, 4–8). As a result, some studies recommend collecting conjunctival samples for surveillance culture studies in the NICU to eradicate these pathogens and prevent sepsis or horizontal transmission of infection (9).

The association between neonatal conjunctivitis or conjunctival discharge and LOS in hospitalized preterm infants has only been reported in only a limited number of literature sources (5, 9–11). Despite the recognized significance of conjunctivitis in NICU settings, there remains a notable gap in understanding its direct relationship with LOS in hospitalized preterm infants. This deficiency in the current literature highlights the necessity of our investigation. Through this retrospective cohort study, we aimed to investigate the relationship between purulent conjunctival discharge and LOS in hospitalized preterm infants. We hypothesized that preterm infants with positive conjunctival swab (CS) cultures were more likely to develop LOS than those with negative culture outcomes.

## Methods

### Study population

We conducted this retrospective cohort study at the Women's Wellness and Research Center (WWRC), formerly the Women's Hospital, part of Hamad Medical Corporation (HMC) in Doha, Qatar. We searched the Microbiology Laboratory database to identify preterm neonates ≤34 weeks who had undergone CS cultures for conjunctivitis between January 2015 and December 2019. These infants are typically admitted to the NICU and often experience prolonged hospital stays. The study group was defined as preterm infants ≤34 weeks of gestation with positive conjunctival swab cultures (CSP). In contrast, the control group includes those with negative conjunctival swab cultures (CSN). Our comparison aimed to elucidate the relationship between CSP and the development of LOS in these hospitalized preterm infants within 120 h of obtaining a CS culture. The 120-hour time frame was chosen as blood cultures may require up to 5 days (or 120 h) to exhibit growth of organisms.

We excluded neonates under the following conditions: positive blood cultures >120 h of obtaining CS culture, neonates born after 34 weeks of gestation, neonates for whom a CS was not obtained, even if they were treated with local or system antimicrobials, CS cultures that grew skin flora or CoNS, CS culture obtained post antimicrobial treatment, and neonates with congenital anomalies.

The study was approved by the WWRC medical research center (MRC), the protocol number (MRC-01-20-329). Due to the retrospective nature of the study, informed consent was not required.

### WWRC overview

WWRC is Qatar's largest governmental tertiary care center, with 82,860 births during the study period. Neonatal practices during the study period included using In-line closed system suctioning, shielding the eyes during suctioning in intubated infants, and sending CSs for Gram stain and bacterial culture for purulent conjunctival discharge cases. Decisions regarding treatment were at the discretion of the physician, with topical antimicrobials usually prescribed until culture results reported.

### Definition

Sepsis in neonates was defined based on two primary criteria. First, neonates who had positive blood cultures (excluding contaminants) obtained within 120 h of taking CS cultures were considered septic. Second, in the absence of a positive blood culture, sepsis was determined by a combination of symptomatic presentation and laboratory findings. Symptomatic presentation included any of the following: new onset desaturations, temperature fluctuations, tachycardia, tachypnea, apnea, feeding intolerance, lethargy, skin mottling, and other clinical conditions deemed by the neonatologist to be suspicious of sepsis, warranting further evaluation. Additionally, laboratory indicators included a high C-reactive protein (CRP) level of ≥10 mg/L and either a platelet count of <100 × 10^3^/ul or an absolute neutrophil count (ANC) of <1.5 × 10^3^/ul.

## Data collection

Our primary objective was to identify late-onset neonatal bacteremia or LOS associated with CSP. Perinatal data were collected by physicians reviewing the electronic patient record, including mothers' charts for clinical and demographic data. Microbiology data was collected from the Microbiology Laboratory's information management system. Perinatal data included birth weight (BW), gestational age (GA), gender, mode of delivery, premature rupture of membranes, chorioamnionitis, Apgar scores, and Group B Streptococcus status. Our secondary objective was to evaluate outcomes associated with CSP cultures. Secondary outcomes included other neonatal infections, the development of new symptoms within 120 h of obtaining CS culture, any new need or unexplained escalation of respiratory support, feeding problems, new onset apnea, death, type of respiratory support, and duration of antimicrobial exposures. Neonatal data were collected from birth and included results of infection markers (WBC counts and CRP), conjunctival swab culture timing and results, blood cultures, and local and systemic antibiotic treatment, as well as clinical outcomes such as type of respiratory support, and feeding problems. We also collected the results of respiratory cultures if done within 2 weeks of CS cultures to determine the association or source of conjunctival bacterial organisms. Where available, the results of respiratory cultures from ventilated babies via tracheal aspirates were collected. The microbiological results were correlated clinically to determine if the organism was a pathogen, colonizer, or contaminant.

## Microbiologic procedures

Conjunctival specimens were taken using an M40 Transystem™ with Amie's agar gel swab (Copan Italia, Brescia, Italy). Swabs were inoculated onto blood, chocolate and MacConkey agar plates, TM (Thayer-Martin agar). Blood and chocolate agar plates were incubated at 35–37°C in 5%–10% CO2 for 24–48 h, and MacConkey agar plates were incubated at 35–37°C for 24 h. Identification and susceptibility testing of all significant organisms was performed using the BD Phoenix™ automated identification and susceptibility testing system (Beckton Dickinson, Franklin Lakes, New Jersey, U.S.A) or for fastidious organisms, using the PhMALDI-TOF mass spectrometry (Bruker, Billerica, Massachusetts, U.S.A) with manual susceptibility testing by ETEST® (bioMérieux, Marcy-l’Étoile, France).

## Statistical analysis

Descriptive analyses were performed for all patient characteristics and clinical variables. Continuous variables are presented as means and standard deviations, and categorical variables are presented as numbers and percentages. The Student *t*-test, *χ*^2^, or Fisher's exact tests were performed, as appropriate, to determine whether variables were different between the two groups (CSP vs. CSN). Logistic regression was applied to examine the associations between various exposures and LOS while controlling for potential confounders. All *p*-values presented were two-tailed, and *p*-values <0.05 were considered statistically significant. All statistical analyses were performed using SPSS for Windows (version 22.0, IBM Corp., Armonk, NY, USA).

## Results

**Figure 1:**
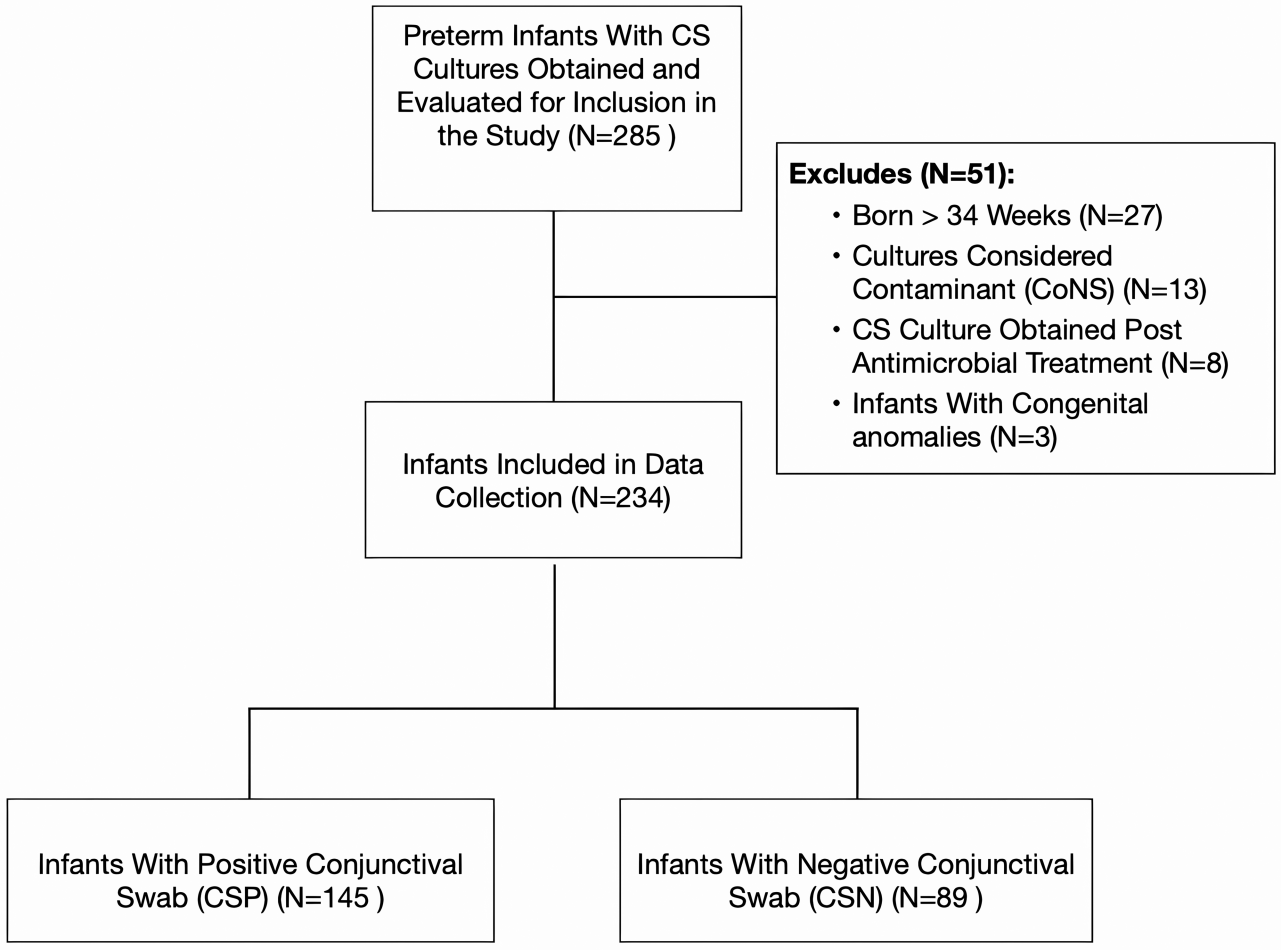
Flow diagram depicting the inclusion criteria and process of conjunctival swab culture collection. CS, conjunctival swab culture; CoNS, coagulase negative staphylococcus.

During the 5-year study period, we identified 285 preterm infants from whom conjunctival swab cultures were obtained (Figure 1). However, several infants were excluded from the study after applying our inclusion criteria. Specifically, 27 infants were above the enrollment GA cutoff, 13 had contaminated CS cultures, eight had undergone a repeat CS culture post antimicrobial treatment for the same condition, and 3 had congenital anomalies. Of the 234 preterm infants analyzed, 145 (61.9%) had positive results (CSP) and 89 (38.1%) yielded negative results (CSN), as outlined in Table 1. On average, eye cultures were taken at 24.5 days across the cohort, with no marked differences between the two groups. Infants in the CSP group had a lower mean BW (1,111 ± 315 g) compared to those in the CSN group (1,230 ± 470 g, *p* = 0.021). They were also younger, averaging 27.9 ± 2.6 weeks gestation against 28.7 ± 3.2 weeks for the CSN group (*p* = 0.032). Furthermore, the CSP group had a lower one-minute APGAR score with 33 infants (22.7%) compared with 9 infants (10.1%) in the CSN group, *p* = 0.014). No significant differences in other baseline demographic variables were observed between the two groups.

**Table 1:** Demographics of preterm infants with and without conjunctival discharge.

Variable	No growth (CSN) *N* = 89	Growth (CSP) *N* = 145	*p*-value
Males	50 (56.2%)	73 (50.3%)	0.386
Vaginal delivery	31 (34.8%)	54 (37.2%)	0.710
PROM	19 (21.3%)	34 (23.4%)	0.709
Apgar 1 min <5	9 (10.1%)	33 (22.7%)	0.014
Apgar 5 min <5	2 (2.2%)	2 (1.4%)	0.636
GA (w)	28.7 ± 3.25	27.9 ± 2.58	0.032
BW (g)	1,230 ± 470	1,111 ± 315	0.021
Eye culture day of life	22.4 ± 19.4	25.7 ± 30.4	0.345
Positive GBS status	9 (14.3%)	19 (15.1%)	0.885
Chorioamnionitis	11 (12.4%)	9 (6.2%)	0.102

GBS, group B streptococci; PROM, premature rupture of membranes; GA, gestational age; BW, birth weight.

Data are *n* (%) or mean (standard deviation) unless otherwise specified, with statistical significance. defined as *P* < 0.05.

Table 2 compares clinical and laboratory variables in preterm infants with CSP to those in the control group. CSP patients were placed on respiratory support more frequently than CSN infants (61.4% vs. 44.9%, *p *= 0.01), primarily (noninvasive ventilation (NIV) (53.8% vs. 36.0%, respectively) and invasive mechanical ventilation (8.3% vs. 9.0%), *p *= 0.001. Neonatal sequential organ failure assessment (nSOFA) was higher in the CSP group (1.64 ± 3.67 vs. 0.13 ± 0.60), *p *< 0.001. Both study and control subjects received topical and systemic antibiotics frequently (97.9% vs. 87.6%, *p *= 0.001, and 38.2% vs. 26.1%, *p *= 0.059, respectively). However, the CSP group had a longer mean duration of antibiotics (topical; 8.9 vs. 5.2 days, *p < *0.001, systemic; 4.1 vs. 1.7 days, *p *< 0.001, respectively). NIV was used by nearly half of all infants (47%), compared to 8.1% on mechanical ventilation and 44.9% on room air. Blood cultures were collected from 41% of all patients, with a higher rate observed in CSP patients (47.5%) compared to CSN patients (33.3%) (*p* = 0.009). Among the CS cultures collected, 7 (10.1%) from the CSP group tested positive, compared to three (11.1%) from the CSN group (*p *= 0.714). In the CSP group, bacteremia with identical CS organisms was identified in 4 patients, constituting 57% of that group. Similarly, LOS was diagnosed in 9 CSP patients (6.2%) compared to 3 (3.4%) CSN patients (*p *= 0.543). LOS was not shown to be a likely outcome in infants with CSP (OR, 1.89 (95% CI, 0.5–7.2, *p *= 0.35). Of the 9 patients who had respiratory cultures performed within 2 weeks of obtaining a CS culture, 5 in the CSP group (5/6 (83%) had the same organisms as in CS cultures.

**Table 2:** Clinical and laboratory characteristics of preterm infants with and without conjunctival discharge.

Variable	No growth (CSN) *N* = 89	Growth (CSP) *N* = 145	*p*-value
New-onset symptoms^a^	19 (21.3%)	45 (31.0%)	0.107
On respiratory support	40 (44.9%)	89 (61.4%)	0.010
Type of respiratory support			0.025
Room air	49 (55.1%)	55 (37.9%)	
Non-invasive	32 (36.0%)	78 (53.8%)	
Invasive	8 (9.0%)	12 (8.3%)	
CRP > 10 mg/L	9 (10.1%)	18 (12.4%)	0.593
Thrombocytopenia	3 (5.2%)	5 (5.0%)	0.999
Low ANC^b^	3 (5.1%)	13 (11.3%)	0.179
nSOFA, score	0.13 ± 0.60	1.64 ± 3.67	<0.001
Topical antibiotics	78 (87.6%)	142 (97.9%)	0.001
Duration of topical antibiotics, days	5.2 ± 3.17	8.9 ± 3.62	<0.001
Systemic antibiotics	23 (26.1%)	55 (38.2%)	0.059
Duration of systemic antibiotics, days	1.69 ± 3.9	4.12 ± 4.5	<0.001
Blood culture obtained	27 (30.3%)	69 (47.6%)	0.009
Bacteremia, any^d^	3 (11.1%)	7 (10.1%)	0.714
Sepsis, any	3.3 (3.4%)	9 (6.2%)	0.543
Respiratory culture sent^c^	3 (3.4%)	6 (4.1%)	0.999

Data are *n* (%) or mean (standard deviation) unless otherwise specified, with statistical significance. defined as *P* < 0.05.

^a^ New-onset symptoms, CRP, C-reactive protein; ANC, absolute neutrophil count; nSOFA, neonatal sequential organ failure assessment.

^b^ Low ANC, <1,500/ul.

^c^ Respiratory cultures were obtained only in intubated neonates.

^d^ Of blood cultures obtained within 120 h in each group.

**Figure 2:**
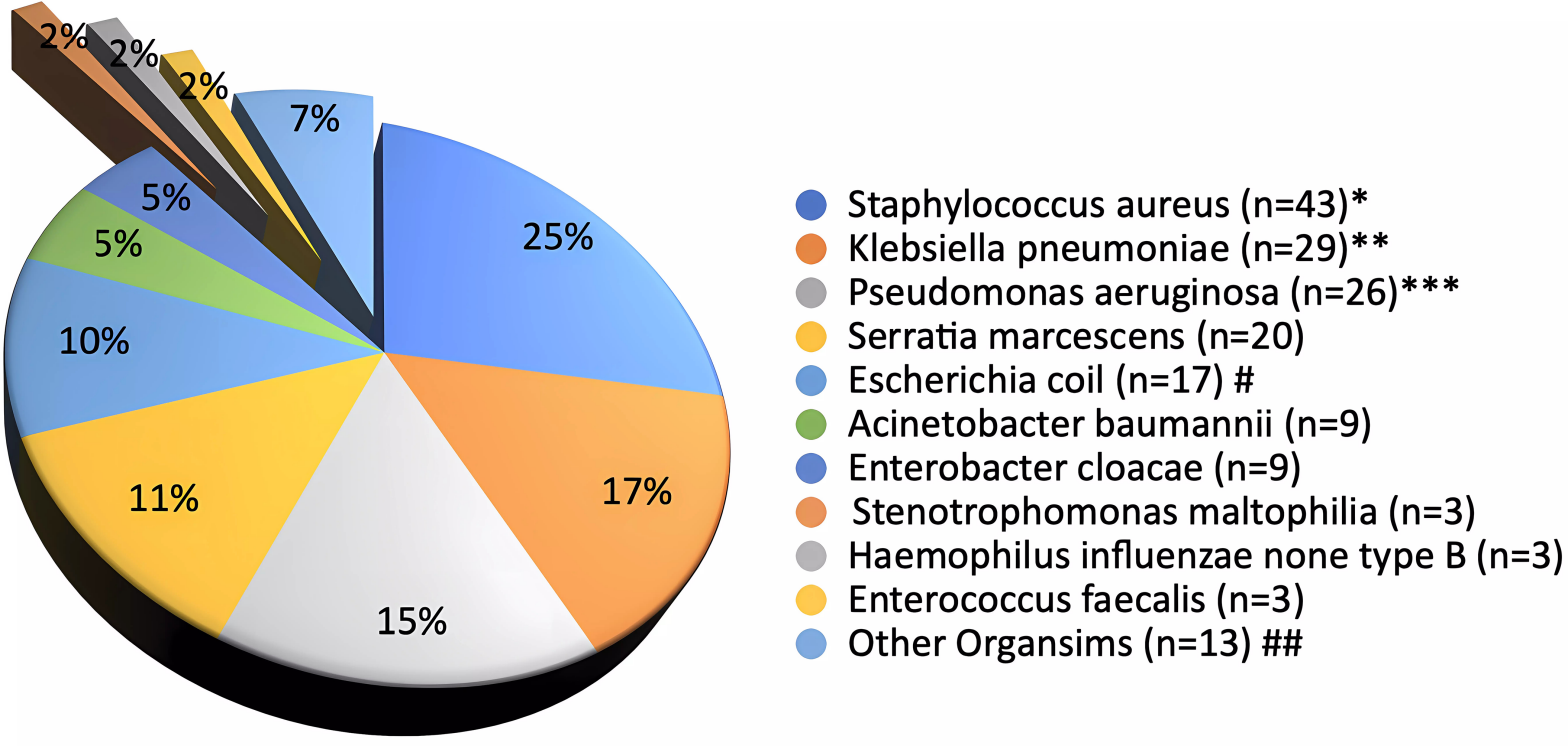
Conjunctival swab culture organisms (*N* = 145 patients and 174 cultures^). ^Cultures grow ≥ two organisms (N=29) *Methicillin-Resistant Staphylococcus aureus (*N*=4/43) **Klebsiella pneumoniae with Extended Spectrum Beta-Lactamase activity (ESBL) (N=3/29) ***Multidrug-Resistant Organisms, P. aeruginosa (*N*=2/26) #ESBL Escherichia coli (*N*=7/17) ##Other organisms: Serratia ureilytica (*n*=2 (1%)), Streptococcus anginosus (*n*=2 (1.1%)), Group B Streptococcus (*n*=2 (1.1%)), Citrobacter koseri *(n*=2 (1.1%)), Streptococcus pneumoniae (*n*=1 (0.6%)), Pseudomonas stutzeri (*n*=2 (0.6%)), Proteus mirabilis (*n*=2 (1%)), Gemella haemolysans (*n*=2 (0.6%)), and Morganella morganii (*n*=2 (0.6%0).

After excluding samples with normal skin flora, Figure 2 lists the isolated 174 organisms from CS cultures sent to 145 CSP patients. Gram-negative organisms accounted for 70% of CS culture results, with gram-positive organisms accounting for the remaining 30%. *Staphylococcus aureus* was the most commonly isolated organism (25%), followed by *Klebsiella pneumoniae* (17%), *Pseudomonas aeruginosa* (15%), *Serratia marcescens* (11%), *E. coli* (10%), *Acinetobacter baumannii* (5%), and *Enterobacter cloacae* (5%). Other Gram-negative bacteria (7%), *Staphylococcus aureus* (3%), and other Gram-positive bacteria (2%). There were 16 resistant organisms (9.2%) among those tested, including 7 extended spectrum beta-lactamase (ESBL) producing *E coli* (43.7%), 4 methicillin-resistant *staphylococcus aureus* (MRSA) (25%), 3 ESBL *Klebsiella pneumoniae* (18.8%), and 2 multi-drug resistant *Pseudomonas aeruginosa* (12.5%). Furthermore, 29 patients had more than one bacterial isolates.

Table 3 presents the organisms identified in both CS and blood cultures of four preterm infants, each exhibiting unique clinical features. A male infant born at 31 weeks, weighing 1,920 grams, developed sepsis due to Serratia marcescens on the 10th day, with the organism identified in CS three days prior. This infant was managed with topical gentamicin sulfate and systemic meropenem. Another male, born at 29 weeks and weighing 960 grams, showed Staphylococcus aureus in his blood culture on day 19, two days following its detection in CS, presenting symptoms like abdominal distension and cellulitis. A female infant born at 31 weeks, weighing 1,440 grams, had the same bacterium identified in her blood culture on day 10, three days prior to its appearance in CS. Treatments for these two infants included gentamicin sulfate, cloxacillin, fusidic acid, and vancomycin. Lastly, a 24-week-old female, weighing 790 grams, exhibited Escherichia coli in her bloodstream on day 1, a day subsequent to its identification in CS. Her treatment comprised gentamicin sulfate and ampicillin, reflecting her mother's cervical infection and her own severe respiratory distress.

**Table 3:** Clinical features and organisms identified in both CS and blood cultures of four preterm infants.

Organism	GA (wks)	BW (g)	Sex	CS culture day	Blood culture day	Associated symptoms	Respiratory Support	CRP	Topical Antibiotics	Systemic antibiotics
Serratia marcescens	31	1,920	Male	10	13	Tachypnea	High flow nasal cannula	8	Gentamicin sulfate for 5 days	Meropenem 10 days
Staphylococcus aureus	29	960	Male	17	19	Abdominal distention, cellulitis and feeding intolerance 3 days post eye discharge	Room Air	5	Gentamicin sulfate for 7 days	Cloxacillin 7 days
	31	1,440	Female	13	10	Fair condition	High flow nasal cannula	6	Fusidic acid for 5 days	Vancomycin 4 days then Cloxacillin 14 days
Escherichia coli	24	790	Female	2	1	Mother with cervical cerclage, cervical culture grew Escherichia coli. Baby with RDS and IVH grade II	Mechanical ventilation	68	Gentamicin sulfate for 7 days	Ampicillin 10 days

CS, conjunctival culture; GA, gestational age; BW, birth weight; RDS, respiratory distress syndrome; IVH, intraventricular hemorrhage; CRP, C-reactive protein.

Using univariate logistic regression analysis, we sought to identify predictors of LOS. Among various significant neonatal and clinical parameters, including CSP, only GA, (marginally significant), the requirement for any form of respiratory support, and invasive ventilation emerged as significant predictors of LOS (Table 4). Subsequently, a multivariate regression analysis, which accounted for both GA and CSP, indicated that infants developing LOS had a higher likelihood of needing invasive respiratory support [adjusted odds ratio (aOR) of 33.5; 95% CI, 2.52–446.5; *p* = 0.008]. The need for continuous positive airway pressure (CPAP) also showed an independent, albeit borderline, association with LOS (aOR, 10.5; 95% CI, 0.94–116.6; *p* = 0.056).

**Table 4:** Univariate and multivariate logistic regression analysis of predictors associated with late-onset neonatal sepsis.

Univariate logistic regression^a^: significant covariates associated with sepsis				
	*p*	OR	95% C.I.	
			Lower	Upper
CSP	0.087	1.71	0.93	3.17
GA	0.052	0.79	0.64	1.00
Any respiratory support*	0.032	9.52	1.21	75.00
Invasive MV	0.002	6.48	2.03	20.71
Multivariate logistic regression^b^: significant covariates on associated with sepsis				
	*p*	OR	95% C.I.	
			Lower	Upper
CPAP	0.056	10.46	0.94	116.56
Invasive MV	0.008	33.55	2.52	446.49

CSP, conjunctival swab positive; GA, gestational age; aOR, adjusted odds ratio; C.I; confidence interval.

^*^ Includes high and low flow nasal cannula; CPAP, continuous positive airway pressure; nasal intermittent ventilation; MV, mechanical ventilation.

^a^ Individual variables were analyzed, and those found to be statistically insignificant (*p* > 0.1) were subsequently excluded from the analysis. These variables include gender, delivery mode, APGAR scores at 1 and 5 min, gestational age (GA), duration of membrane rupture, CS culture day, Group B Streptococcus status, chorioamnionitis, and multiple gestation.

^b^ Controlled for GA and CSP.

## Discussion

Purulent conjunctival discharge in preterm infants may pose a clinical quandary in terms of approach and relationship to the development of sepsis, as it may indicate an underlying infection. The high prevalence of positive results for CSP among the analyzed preterm infants, accounting for nearly 62%, indeed warrants a closer examination of the clinical implications and underscores the importance of heightened clinical vigilance in the NICU setting. This finding highlights the significance of conjunctival discharge as a potential reservoir for bacterial pathogens in these vulnerable neonates especially those on respiratory support. Despite the practice of covering the eyes with multilayer gauze during mouth and airway suctioning throughout the study years, a significant number of patients required evaluation for eye discharge, as indicated by the study. Among the patients, blood cultures were obtained in 41.5%, while CRP and complete blood count tests were conducted for 60% within 5 days of the onset of eye discharge. Furthermore, topical antibiotics were prescribed and continued in the vast majority of cases, and systemic antibiotics were initiated in one-third of all cases while awaiting the results of CS cultures and continued in a significant number of CSP patients, raising clinical concerns regarding effective antimicrobial stewardship in the NICU. It should be noted, however, that the level of compliance with the use of multilayer gauze during suctioning is not known. Therefore, there could be an increased risk of exposure to pathogens from the respiratory secretions, potentially contributing to a higher incidence of purulent eye discharge. The presence of new-onset symptoms associated with eye discharge, on the other hand, was not a statistically significant finding.

Although *Staphylococcus aureus* was the most commonly isolated organism, Gram-negative organisms overall made up more than two-thirds of the isolated organisms, particularly *Klebsiella pneumoniae, Pseudomonas aeruginosa, Serratia marcescens, and E. coli*. Furthermore, resistant strains accounted for approximately 9% of the organisms in the entire cohort. The findings of this bacterial pattern in our study, especially the predominance of Staphylococcus spp., are consistent with published surveillance and clinical reports on conjunctivitis in neonates admitted to the NICU (1, 12–14), where the most common pathogens were coagulase-negative staphylococci (25%), Staphylococcus aureus (19%), and Klebsiella spp. (10%).

According to the findings, risk factors for conjunctivitis include low BW and younger GA, a low 1-minute APGAR score, and NIV (Tables 2, 3). Consistent with other studies' findings, these infants are typically the ones who require extended periods of respiratory support and recurrent ophthalmologic evaluations (1). However, as the study indicates, we were unable to demonstrate a significant independent correlation between LOS and conjunctival discharge in these infants, implying that conjunctival discharge may not be a reliable predictor of LOS in premature infants admitted to the NICU. However, as the study suggests, among other variables in these patients, sepsis is primarily associated with invasive ventilation, whereas NIV is insignificant.

As previously stated, the study found a correlation between conjunctival discharge in hospitalized preterm infants and respiratory support, namely NIV. Respiratory secretions may be transferred from the nasopharynx to the eyes during suctioning, which is required for infants who require ventilatory assistance (1). In a study of a *P. aeruginosa* conjunctivitis outbreak in a pediatric hospital, 70% of patients with respiratory cultures obtained before the onset of conjunctivitis were colonized with this organism (15). This suggests that respiratory secretions could be the source of infection in these case. However, it is important to note that surveillance CS cultures are rarely performed in most NICUs nowadays because positive culture results in these infants have always been considered colonization from the respiratory tract if not associated with other clinical manifestations of conjunctivitis. As a result, these surveillance cultures are regarded as having little utility in the diagnosis of conjunctivitis (2). Furthermore, many cases of conjunctivitis in hospitalized preterm infants fail to meet the NNIS definition, partly due to clinicians' attitude toward eye discharge and the tendency to begin empiric treatment with or without sending CS cultures, and partly due to the absence of other clinical manifestations in preterm infants, which causes the NNIS to underestimate a significant number of cases.

Due to the retrospective nature of our study, the reliability of electronic patient records may present challenges. Potential issues include data entry errors, incomplete or inconsistent records, data loss during system upgrades, and biases in data recording. Additionally, older non-digitized records may be omitted, affecting the dataset's comprehensiveness and accuracy. It's crucial to consider these factors when interpreting the findings. Moreover, while conjunctival discharge is a recognized sign of conjunctivitis in preterm patients, there were inconsistencies in how other clinical signs were documented, complicating retrospective data retrieval. In this study, the choice to perform blood cultures in the face of conjunctival discharge varied by physician, reflecting the absence of well-established guidelines on this issue. Distinguishing between colonization and genuine conjunctivitis presents another challenge that could affect our conclusions. Additionally, sole reliance on the Microbiology Laboratory database also brings its own set of biases related to sample collection, culturing, and automated system limitations. Cross-referencing clinical data and using diverse growth mediums is crucial. Notably, while invasive ventilation is found to be a risk factor for LOS in those infants with positive CS, in most cases we are unable to confirm the presence of the same organism in respiratory samples of positive CS cultures. Furthermore, the study was conducted in a single NICU and the findings may not be generalizable to other settings.

The study sheds light on the potential relationship between the risk of LOS in ventilated preterm infants with purulent conjunctival discharge and emphasizes the importance of careful monitoring in this vulnerable population. Despite the limitations and the data presented, the potential role of respiratory secretions in neonatal conjunctival infection or colonization underscores the importance of employing proper techniques and stringent infection control measures during airway suctioning.

## Conclusion

This data suggests that positive CS is common in hospitalized preterm infants with purulent eye discharge, particularly those on NIV. Although no statistically significant correlation was found between purulent conjunctivitis with a positive CS culture and LOS, the mere finding of a common pathogen isolated in both eye discharge and blood cultures in many cases signifies that there is indeed a need for further investigation in wider populations. Future studies with comprehensive documentation of clinical signs can enhance the generalizability of these findings. Such studies could further explore the relationship between purulent conjunctival discharge and LOS in ventilated preterm infants. The findings could potentially contribute to the development of evidence-based guidelines for managing conjunctival discharge in this population. Furthermore, these studies may emphasize the importance of judicious antibiotic prescribing practices in the NICU. Additionally, research focused on evaluating the effectiveness of prophylactic measures, such as eye care, in reducing the incidence of LOS in ventilated premature infants with conjunctival discharge should be considered.

## Data Availability

The raw data supporting the conclusions of this article will be made available by the authors, without undue reservation.
